# Parahydrogen-induced polarization with a metal-free P–P biradicaloid†
†Electronic supplementary information (ESI) available: Description of the experimental procedure, and additional NMR spectra illustrating results of the work. See DOI: 10.1039/c8cp07625a


**DOI:** 10.1039/c8cp07625a

**Published:** 2019-01-22

**Authors:** Vladimir V. Zhivonitko, Jonas Bresien, Axel Schulz, Igor V. Koptyug

**Affiliations:** a NMR Research Unit , University of Oulu , P.O. Box 3000 , 90014 Oulu , Finland . Email: vladimir.zhivonitko@oulu.fi; b University of Rostock , Institute of Chemistry , Albert-Einstein-Str. 3a , 18059 Rostock , Germany; c Leibniz-Institut für Katalyse e.V. an der Universität Rostock , Albert-Einstein-Strasse 29a , 18059 Rostock , Germany; d Laboratory of Magnetic Resonance Microimaging , International Tomography Center SB RAS , Instututskaya St. 3A , 630090 Novosibirsk , Russia; e Novosibirsk State University , Pirogova St. 2 , 630090 Novosibirsk , Russia

## Abstract

Metal-free H_2_ activations are unusual but interesting for catalytic transformations, particularly in parahydrogen-based nuclear spin hyperpolarization techniques. We demonstrate that metal-free singlet phosphorus biradicaloid, [P(μ-NTer)]_2_, provides pronounced ^1^H and ^31^P hyperpolarization while activating the parahydrogen molecules. A brief analysis of the resulting NMR signals and the important kinetic parameters are presented.

## Abstract 2

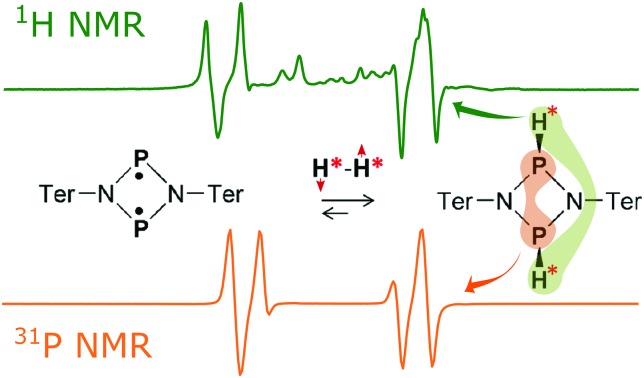
The activation of parahydrogen by a metal-free P–P biradicaloid leads to ^1^H and ^31^P nuclear spin hyperpolarization.

## 

Metal-free activation of small molecules like dihydrogen attracts a lot of attention, especially in the context of the development of novel and sustainable catalytic systems.^1^ On the other hand, it is highly interesting for boosting sensitivity in nuclear magnetic resonance (NMR). Breaking the symmetry of H_2_ in chemical transformations is at the heart of nuclear spin hyperpolarization techniques such as parahydrogen-induced polarization (PHIP)^2^ and signal amplification by reversible exchange (SABRE),^3^ which can be used to enhance the NMR signals by orders of magnitude. Parahydrogen is a nuclear spin isomer of H_2_ which accommodates only nuclear spin singlets. It is easily accessible *via* an *ortho*–*para* conversion process at low temperatures, serving as a source of non-equilibrium nuclear spin order. Typically, metal-based catalysts (transition metal complexes,^4^ metal nanoparticles,^5^ metal oxides^6^) are used to generate hyperpolarized substances. In many cases, a limiting factor for the practical applications of this technique is the presence of the toxic metal catalysts along with the hyperpolarized product in the reaction mixture, and their separation is not a trivial task. Several approaches have been tested to achieve separation, including hydrolysis/extraction of the hyperpolarized product in the case of homogeneous hydrogenation catalysts,^7^,^8^ and the utilization of heterogeneous catalysts which, in addition to supported metals and metal oxides mentioned earlier, include surface-immobilized metal complexes^9^–^13^ and catalytically active isolated surface sites and single-atom catalysts.^14^–^17^ In this respect, the rather unusual metal-free activations of H_2_ can be very interesting for providing metal-free hyperpolarized agents for *in vivo* use and other applications of NMR and MRI.

So far, several metal-free systems have demonstrated nuclear spin hyperpolarization effects upon activation of parahydrogen molecules.^18^–^21^ They can be divided into two groups: one includes various ansa-aminoboranes^18^–^20^ while the other presents a single instance of an aromatic phosphabenzene.^21^ Ansa-aminoboranes, referred to as molecular tweezers,^22^ activate parahydrogen reversibly under mild conditions (room temperature), which leads to continuous hyperpolarization of ansa-aminoborane–H_2_ adducts after each fresh portion of parahydrogen is introduced. In a recent publication, it was shown that in addition to ^1^H nuclei the ^15^N nuclear spins are also substantially polarized as a result of the chemical activation of parahydrogen.^20^ Moreover, ^15^N nuclei of both free ansa-aminoborane and its H_2_ adduct were hyperpolarized, constituting the first example of hyperpolarization without actual chemical modification, which is the specific feature of the SABRE technique. In the case of the aromatic phosphabenzene,^21^ the activation of parahydrogen led to hyperpolarization of ^1^H nuclei of the resulting adduct molecules at 383 K.

Both the ansa-aminoboranes and the aromatic phosphabenzene activate H_2_ molecules by the frustrated Lewis pairs (FLP),^23^ the N–B and P–C pairs that these compounds possess, respectively, as part of their structure. Other classes of metal-free H_2_ activators such as selected carbenes,^24^ digermynes^25^ and biradicaloids^26^ may be of potential interest for parahydrogen-based hyperpolarization techniques. In particular, the recent results on the open-shell singlet diphosphadiazanediyl biradicaloid, [P(μ-NTer)]_2_ (Ter = 2,6-Mes_2_–C_6_H_3_),^27^ have demonstrated that this compound easily activates dihydrogen under ambient conditions (Scheme 1), forming exclusively the thermodynamically and kinetically preferred *cis* isomer in accordance with theory (Δ_298_*G*_*cis*–*trans*_ = 2.7 kcal mol^–1^, see ESI†). Since the two electron spins of the biradicaloid form a singlet spin state, we expect that addition of H_2_ to the biradicaloid would be akin to H_2_ addition to a multiple bond of a closed-shell molecule.

**Scheme 1:**
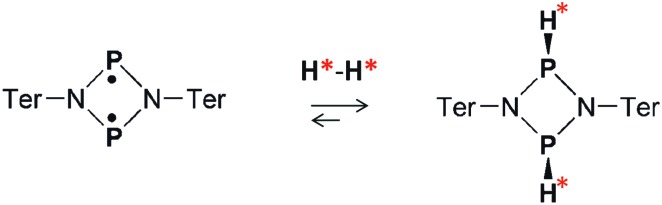
Dihydrogen activation with [P(μ-NTer)]_2_ biradicaloid.

Herein, we present a study of [P(μ-NTer)]_2_ in the context of parahydrogen activation and observation of nuclear spin hyperpolarization effects. We demonstrate that the biradicaloid can be used to hyperpolarize ^1^H as well as ^31^P nuclei in this process. The reaction kinetics parameters are measured to quantify the rate of *cis*-[HP(μ-NTer)]_2_ adduct dissociation, and explain the observed hyperpolarization effects at high and low reaction temperatures.

In the first experiments, we have tested the hyperpolarization effects observed after parahydrogen bubbling through a 0.04 M solution of [P(μ-NTer)]_2_ at 293 K and *ca.* 1 bar absolute pressure. Only a few bubbling repetitions were possible before the full conversion of the biradicaloid to the H_2_ adduct was reached under these experimental conditions. Importantly, hyperpolarization effects were immediately observed in the ^1^H NMR spectra after the first bubbling until the moment of complete consumption of the initial biradicaloid. Fig. 1a shows a representative ^1^H NMR spectrum. See also Fig. S1 in the ESI† for the spectra of the full ppm range.

**Fig. 1:**
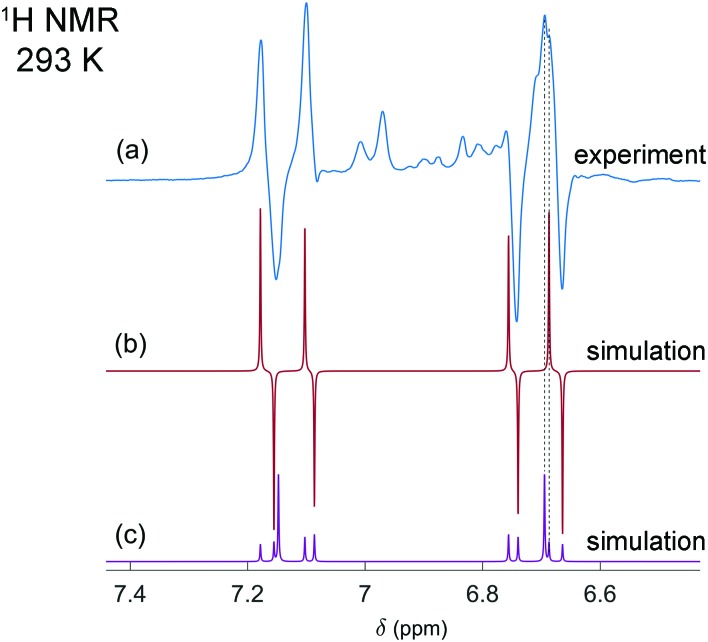
Experimental (a) and simulated (b and c) ^1^H NMR spectra of the [HP(μ-NTer)]_2_ adduct. The experimental spectrum (a) is acquired after parahydrogen bubbling through a 0.04 M solution of the biradicaloid at 293 K and 1 bar pressure. The simulation in (b) is based on the assumption that in the experiment the H–H pair in [HP(μ-NTer)]_2_ was initially in the singlet nuclear spin state originating from parahydrogen. The simulated spectrum (c) corresponds to the thermally equilibrated spin state. Arbitrary intensity scales are used to draw each spectrum. The black dashed lines show correspondence between the lines in the simulated and in the experimental spectra.

The observed enhanced multiplet has a complex structure of absorption and emission lines, which does not look like the usual PASADENA-type antiphase pattern, which is commonly observed when reaction with parahydrogen takes place in a high magnetic field.^28^ The reaction results in an AA′XX′ spin system, wherein the H–H and P–P spin pairs are strongly coupled. The numerical simulations of multiplet patterns, expected theoretically for the hyperpolarization and the thermal polarization states, are shown in Fig. 1b and c, respectively (see also ESI† for details). The hyperpolarization patterns in the simulation and the experiment do not completely match each other. In particular, the lines with the largest amplitude in the thermal spectrum are not expected to show any polarization according to the simulated spectrum since they correspond to the triplet spin states, specifically T_+_ and T_–_, of the H–H pair (see Fig. S3 in ESI†). Normally, such states should not be produced because the chemical activation is not expected to induce the transition of the purely singlet nuclear spin state (S) stemming from the parahydrogen to those triplet ones. Only S and T_0_ states can be mixed. However, the experimentally detected spectra do show considerable enhancement for these lines. Likely, the observation of the hyperpolarization in the H–H triplet domain indicates transitions induced by the nuclear spin relaxation or possibly some complex spin dynamics, which involves free electrons. More details are present in the ESI.† The full theoretical understanding of this phenomenon, however, is outside the scope of this communication and will be studied separately.

It is interesting that the activation of parahydrogen, which consists of ^1^H nuclei only, leads also to the hyperpolarization of ^31^P nuclei in the resulting molecularly symmetric *cis*-[HP(μ-NTer)]_2_. Fig. 2a and b show the experimental spectra detected after the parahydrogen bubbling and after the full relaxation to thermal equilibrium, respectively. The enhanced ^31^P multiplet has a shape similar to that observed in ^1^H NMR spectra. The same can be noticed in the case of thermal polarization; compare Fig. 1c and 2b. It happens due to the strong coupling in the H–H and P–P pairs and the presence of some coupling between the pairs. In this situation, the spins behave like there are no individual spins but rather their superpositions (see ESI† for details). Then, if one part of the entire spin system is hyperpolarized (^1^H pairs), it is also visible in another part (^31^P pairs). The estimation of the signal enhancements was easy to perform for ^31^P, because there are no other background signals masking the weaker thermal signals. The measured enhancements were in the range from 60 to 300 depending on which particular line is considered.

**Fig. 2:**
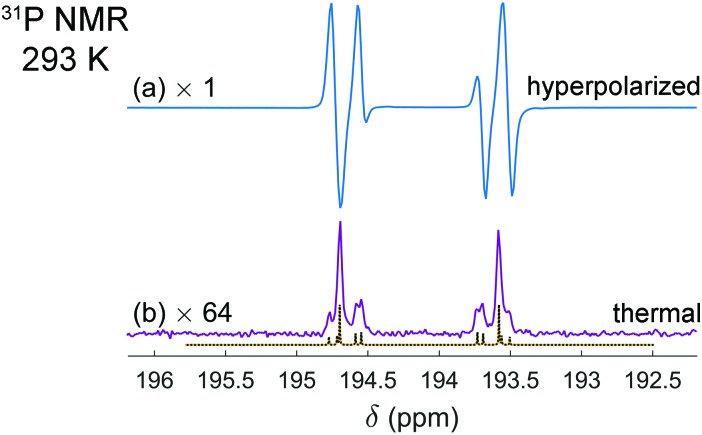
Experimental ^31^P NMR spectra of the [HP(μ-NTer)]_2_ adduct acquired after parahydrogen bubbling (a) and after the relaxation to thermal equilibrium (b). The simulated thermal spectrum is added to (b) for comparison. The same experimental conditions were used as described in Fig. 1 caption. The spectrum (b) is multiplied by the factor of 64 for the sake of better visualization. The spectra reveal the signal enhancement on the order of 60 to 300, depending on the particular spectral line.

The reaction at 293 K allowed us to observe the hyperpolarization effects for [HP(μ-NTer)]_2_ only within a short time frame when some amount of free [P(μ-NTer)]_2_ was still available to perform the activation. After the full conversion, the polarization effects disappeared completely. We note, however, that upon heating the sample to 353 K we observe the polarized signals repeatedly after every bubbling of parahydrogen (Fig. S2 in ESI†). The enhanced signals observed in both ^1^H and ^31^P NMR spectra were similar to those described above. It is highly likely that this implies that the reaction becomes reversible enough for the new portions of parahydrogen to react with a considerable fraction of available free [P(μ-NTer)]_2_. This means that effectively the elevated temperature allows the replacement of the relaxed hydrogens in the [HP(μ-NTer)]_2_ within a shorter time frame than that needed for the full relaxation of the hyperpolarization. Similar behavior was observed in the previous studies with ansa-aminoboranes.^18^–^20^

In order to understand the dissociation kinetics of [HP(μ-NTer)]_2_, which is responsible for the formation of free [P(μ-NTer)]_2_, we have measured the kinetic constants using the spin saturation transfer method, and also have determined the activation energy of the dissociation process (Fig. 3 and ESI†).

**Fig. 3:**
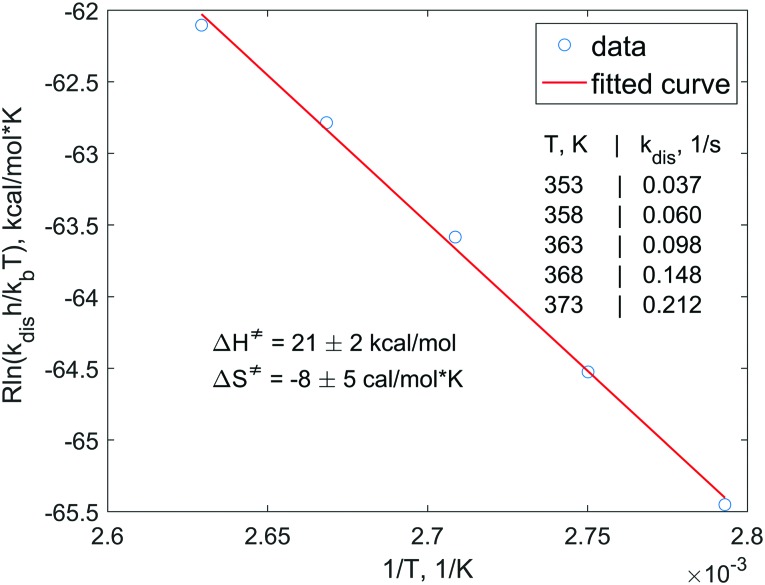
Eyring plot for the [HP(μ-NTer)]_2_ dissociation process. Experimental points are drawn with circles and the linear fitting result is shown with a red line. Experimentally measured rate constants and the activation parameters obtained from the fitting at 95% confidence level are also shown in the graph.

According to this data, the characteristic exchange time for the H atoms (1/*k*_dis_) is *ca.* 27 s at 353 K. This parameter is considerably longer than the corresponding relaxation time of, *e.g.*, ^31^P nuclei (*ca.* 1 s). Also, the activation energies (Δ*G*‡298 = Δ*H*^‡^ – *T*Δ*S*^‡^) of the dissociation are considerably higher (*ca.* 23 kcal mol^–1^, calcd. 24.4 kcal mol^–1^, see ESI†) as compared, *e.g.*, to those observed in reversible activation of parahydrogen with ansa-aminoboranes (*ca.* 18 kcal mol^–1^).^19^,^29^ Altogether, this shows that [P(μ-NTer)]_2_ is a good but not the best candidate for providing the highest hyperpolarization levels in the reversible activation mode since it takes a relatively long time before the adduct dissociates to be able to accept a fresh portion of parahydrogen. On the other hand, its structure can be optimized to provide the desired kinetic properties.

As the biradicaloid is in the singlet electronic state, we do not expect any effect of unpaired electrons on the observed hyperpolarization. Dealing with radicals, however, care must be taken to exclude the possibility that the hyperpolarization effects are generated by a free radical mechanism such as the chemically induced dynamic nuclear polarization (CIDNP). In this phenomenon, the electron spins participate in the formation of the hyperpolarized state. To prove that potential formation of radical pairs in the process of H_2_ activation is not responsible for the hyperpolarization effects reported here, we performed experiments using normal H_2_ instead of parahydrogen. This test has unambiguously shown that normal H_2_ does not lead to hyperpolarization effects, whereas parahydrogen does, proving the PHIP-based mechanism of the hyperpolarization.

Using the principles of spin-lock induced crossing (SLIC),^30^ the observed complex ^1^H or ^31^P multiplets consisting of multiple absorption and emission lines can be transformed into strongly enhanced in-phase signals by applying long ^1^H or ^31^P radio frequency pulses of low power followed by the detection with ^31^P or ^1^H decoupling, respectively. The enhanced in-phase signals may be more suitable, for instance, in imaging experiments. An example of such an SLIC experiment for ^31^P resonances is presented in Fig. S4 (ESI†).

## Conclusions

In conclusion, we have demonstrated that the use of [P(μ-NTer)]_2_ biradicaloid leads to the strong hyperpolarization effects detected in ^1^H and ^31^P NMR spectra in the parahydrogen activation process. It is the first time, in general, that a metal-free compound of a biradicaloid structure is shown to produce hyperpolarized nuclear spins, introducing a new class of parahydrogen activators for NMR signal enhancement. This observation also proves that the mechanism of H_2_ activation with [P(μ-NTer)]_2_ is pairwise, meaning that the two hydrogen atoms of an H_2_ molecule follow each other throughout all the elementary stages of the activation process. As strong as 300-fold signal enhancement was detected in ^31^P NMR spectra of [HP(μ-NTer)]_2_ without any additional manipulations with magnetic fields, which are commonly required to transfer ^1^H hyperpolarization to heteronuclei. It takes place as a result of the formation of an AA′XX′ spin system made out of H–H and P–P pairs in the symmetric adduct. The hyperpolarization transfer from ^1^H to ^31^P happens naturally *via* a coherent process mediated by the *J*-coupling network. Therefore, [HP(μ-NTer)]_2_ is an interesting model molecule for probing the spin dynamics of symmetric molecules. Moreover, the strong NMR signals from ^31^P have the advantage of insensitivity to the presence of ^1^H-containing molecules in a sample under study, which is useful, for instance, in NMR imaging (or MRI) to get rid of background signals. We have also shown that the initial complex ^31^P multiplet can be converted to an in-phase signal by using the SLIC technique,^29^ allowing the use of standard NMR spectroscopy or imaging methods in studies involving hyperpolarized [HP(μ-NTer)]_2_. We also believe that new structural/mechanistic details can be uncovered by detailed NMR studies of this molecule, as suggested, for instance, by the lack of the full correspondence between the theoretical and experimental spectra (Fig. 1).

This work was supported by the University of Oulu (Kvantum Institute), the European Research Council (ERC) under Horizon 2020 (H2020/2018-2022/ERC Grant Agreement No. 772110) and the SB RAS integrated research program (# 0333-2018-0006/II.1.13). IVK thanks the Russian Ministry of Science and Higher Education (# 0267-2019-0004) for financial support.

## Conflicts of interest

There are no conflicts to declare.

## Supplementary Material

Supplementary information
