# Supplementary material for: Parahydrogen-induced polarization with a metal-free P–P biradicaloid
Source: Phys Chem Chem Phys. 2019 Jan 22;21(11):5890–3. doi: 10.1039/c8cp07625a (PMC6430094; doi:10.1039/c8cp07625a)
Supplement: Supplementary file 1 [file CP-021-C8CP07625A-s001.pdf]

Electronic Supplementary Material (ESI) for Physical Chemistry Chemical Physics.
This journal is © the Owner Societies 2019

Supporting Information

Parahydrogen-induced polarization with a metal-free P-P biradicaloid

Vladimir V. Zhivonitko,*[a] Jonas Bresien, [b] Axel Schulz [b] and Igor V. Koptyug [c,d]

[a.] NMR Research Unit, University of Oulu, P.O. Box 3000, 90014 Oulu (Finland), E-mail: vladimir.zhivonitko@oulu.fi.

[b.] Leibniz-Institut für Katalyse e.V. an der Universität Rostock, Albert-Einstein-Strasse 29a, 18059 Rostock (Germany).

[c.] Laboratory of Magnetic Resonance Microimaging, International Tomography Center SB RAS, Institutskaya St. 3A, 630090 Novosibirsk (Russia).

[d.] Novosibirsk State University, Pirogova St 2., Novosibirsk 630090, Russia

-CONTENTS-

1 Materials and experimental methods 2
1.1 General information 2
1.2 NMR experiments 2
1.2.1 Full range ^{1}H spectra acquired at 293 K 2
1.2.2 ^{31}P NMR spectra acquired at 353 K after parahydrogen bubbling 3
1.2.3 Simulations of NMR spectra of [HP(μ-NTer)]$_2$ 4
1.2.4 Spin states of AA'XX' spin system of H-H and P-P pairs in [HP(μ-NTer)]$_2$ 5
1.2.5 Kinetic measurements 6
1.2.6 SLIC spectra 7
2 Computational details 8
3 References 9

Figure S1. ^{1}H NMR spectra obtained after a 5 s parahydrogen bubbling through a 0.04 M toluene-d_8 solution of [P(μ-NTer)]$_2$ (a), and after the relaxation to thermal equilibrium (b) at 293 K. Both spectra were acquired using π/4-pulses. ...3
Figure S2. Experimental ^{31}P NMR spectra of adduct [HP(μ-NTer)]$_2$ acquired after parahydrogen bubbling at 353 K through the equilibrium solution after the full conversion of [P(μ-NTer)]$_2$ (a), and after the relaxation of the nuclear spins (b). The spectrum (b) is multiplied by a factor of 32 relative to spectrum (a). The comparison of the spectra reveals the signal enhancement on the order of magnitude from 60 to 300, depending on a transition line. Both spectra were acquired using π/4-pulses. The signal-to-noise ratio was lower at 353 K as compared to 293 K (Figure 2b of the main text) due to the sensitivity of ^{31}P RF probe to the temperature. 4
Figure S3. A simulated thermal ^{1}H NMR multiplet corresponding to H_2 originating atoms in [HP(μ-NTer)]$_2$ adduct (blue trace) and all possible non-zero contributions of the AA'XX' spin states to the multiplet's most intense transition lines (red, yellow, violet, green traces). See the legend for the contributing spin states. 6
Figure S4. (a) Experimental ^{1}H decoupled ^{31}P NMR spectrum obtained by applying a weak-power (63 Hz RF field amplitude) 42 ms pulse after parahydrogen bubbling. (b) ^{1}H decoupled ^{31}P NMR spectrum obtained by using a hard π/4-pulse after the relaxation of spins to thermal equilibrium. The spectrum (b) is multiplied by a factor of 32 relative to spectrum (a). The small "satellite" signals in (a) are artifacts of non-ideal ^{1}H decoupling. 8
Scheme S1. Pulse sequence employed to perform ^{31}P SLIC experiments with ^{1}H decoupling. 7

1 Materials and experimental methods

1.1 General information

NMR experiments with parahydrigen were performed on a 300 MHz Bruker AV 300 NMR spectrometer equipped with a broad-band 10 mm RF probe. The standard temperature control unit of the NMR spectrometer was used for cooling and heating the samples. The kinetic measurements were performed on a 400 MHz Spectrometer Bruker AV 400 NMR equipped with a broad-band 5 mm RF probe.

$[P(\mu\text{-NTer})]_2$ was synthesized using procedures described in Refs.[S1,2]

High-purity commercially available H_2 gas was used for producing parahydrogen-enriched H_2 referred to in the main text as simply parahydrogen. The enrichment was performed with a Bruker parahydrogen generator, which produced H_2 gas with 91 % of parahydrogen.

1.2 NMR experiments

In a typical workflow, parahydrogen was bubbled trough a 0.04 M solution of the biradicaloid $[P(\mu\text{-NTer})]_2$ in degassed toluene-d_8 in a 5 mm sample inside the NMR magnet for ca. 5 s, and then the parahydrogen flow was abruptly switched off and an NMR experiment was started. The bubbling procedure was performed under atmospheric pressure (1 bar) in the same manner as explained in detail in Ref.[S3] Parahydrogen was supplied to the bottom of the sample tube through a 1/32" PTFE tubing. The sample temperature was varied in the experiments when it was required.

Since $[P(\mu\text{-NTer})]_2$ is highly sensitive to both air and moisture, the sample preparation procedures were done under inert Ar atmosphere.

1.2.1 Full range ^1H spectra acquired at 293 K

A full-range ^1H NMR spectrum acquired after parahydrogen bubbling through the 0.04 M solution of $[P(\mu\text{-NTer})]_2$ in toluene-d_8 is shown in Figure S1a, with part of this spectrum also presented in Figure 1a. For comparison, a thermal equilibrium spectrum after the relaxation is shown in Figure S1b. The detection of unusual antiphase signals after the parahydrogen bubbling serves as a solid justification for the formation of the hyperpolarized state.

The P-P and H-H pairs form the symmetric AA'XX' spin system in the resulting $[HP(\mu\text{-NTer})]_2$ adduct. Nevertheless, like in common PASADENA experiments,[S4] $\pi/4$ RF pulses provided higher signal amplitudes compared to those obtained with $\pi/2$-pulses. The exact ^1H signal enhancement measurement was difficult to perform, because thermally polarized signals of the adduct $[HP(\mu\text{-NTer})]_2$ were hampered by the signals of aromatic groups in the measured ^1H NMR

spectra. The more precise estimation of enhancement was done using ^{31}P NMR spectra as described in the main text.

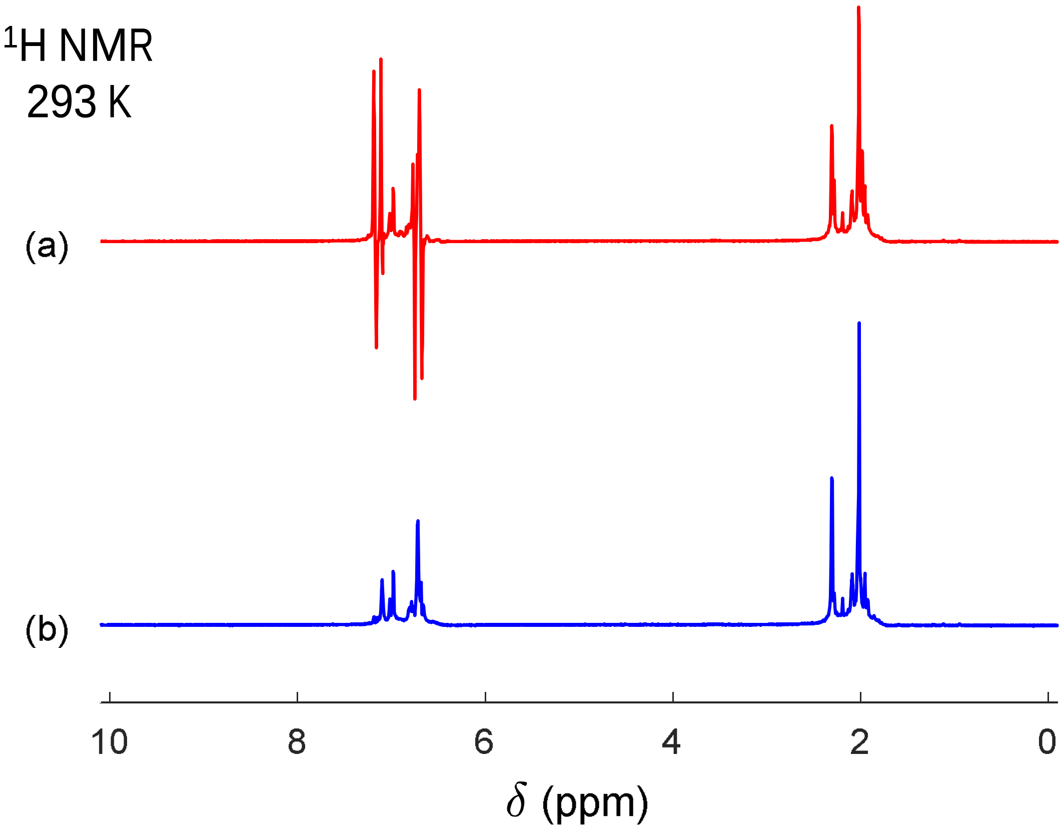

Figure S1. ^1H NMR spectra obtained after a 5 s parahydrogen bubbling through a 0.04 M toluene-d_8 solution of [P(μ-NTer)]$_2$ (a), and after the relaxation to thermal equilibrium (b) at 293 K. Both spectra were acquired using π/4-pulses.

1.2.2 ^{31}P NMR spectra acquired at 353 K after parahydrogen bubbling

As described in the main text, the polarized signals begin to appear after every bubbling of parahydrogen upon heating the sample to 353 K, while at 293 K polarization effects are no longer visible after a few bubbling repetitions because of the full conversion of [P(μ-NTer)]$_2$ to [HP(μ-NTer)]$_2$. This implies that the reaction at this temperature becomes reversible "enough" so that parahydrogen can replace the "relaxed" hydrogens in [HP(μ-NTer)]$_2$. Figure S2a shows ^{31}P NMR spectra observed reproducibly after the parahydrogen bubbling at 353 K through the reaction mixture. The ^{31}P signal multiplet measured at 353 K (Figure S2a) looks very similar to that measured at 293 K (Figure 2a of the main text). The corresponding thermal spectrum is depicted in Figure S2b. It should be noted that mainly due to the sensitivity of the ^{31}P RF probe to the temperature in our setup, the thermal polarization ^{31}P signal at 353 K (Figure S2b) was weaker as compared to that at 293 K (Figure 2b).

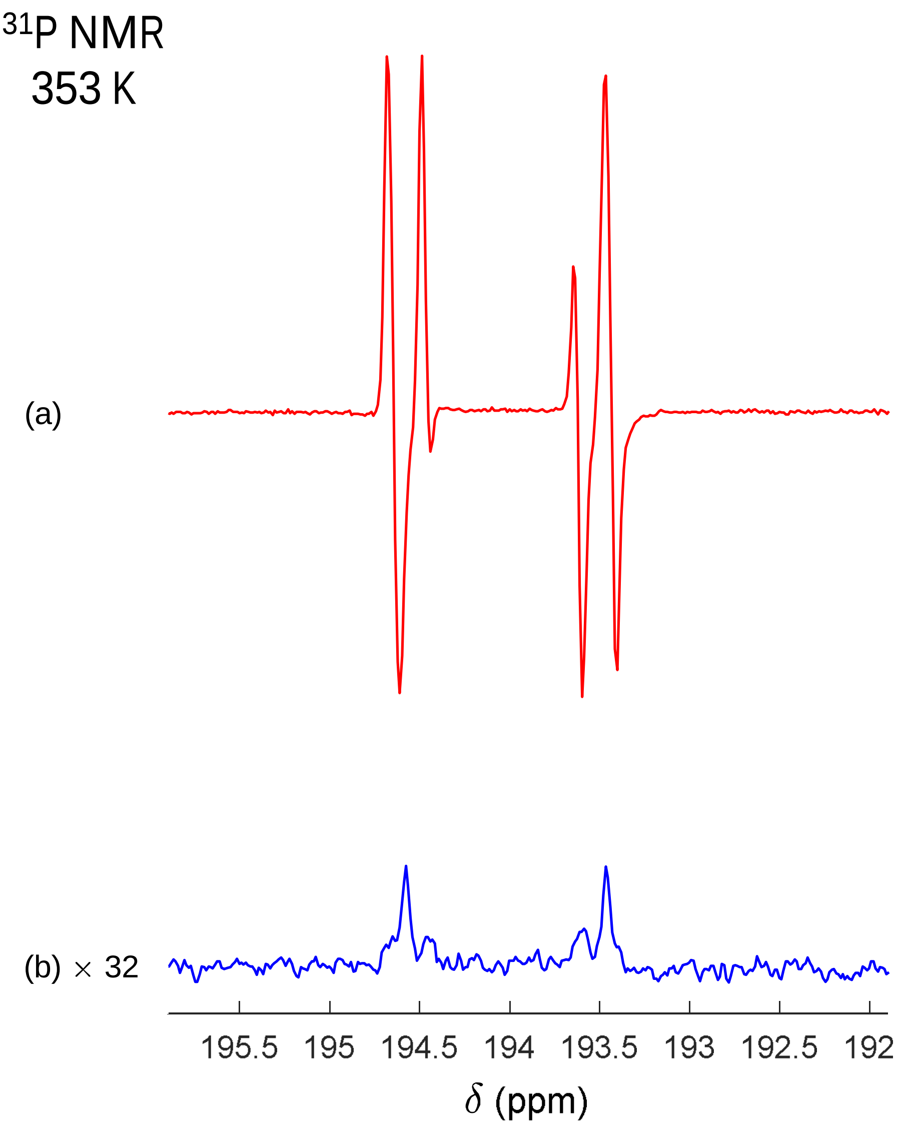

Figure S2. Experimental ^{31}P NMR spectra of adduct [HP(μ-NTer)]$_2$ acquired after parahydrogen bubbling at 353 K through the equilibrium solution after the full conversion of [P(μ-NTer)]$_2$ (a), and after the relaxation of the nuclear spins (b). The spectrum (b) is multiplied by a factor of 32 relative to spectrum (a). The comparison of the spectra reveals the signal enhancement on the order of magnitude from 60 to 300, depending on a transition line. Both spectra were acquired using π/4-pulses. The signal-to-noise ratio was lower at 353 K as compared to 293 K (Figure 2b of the main text) due to the sensitivity of ^{31}P RF probe to the temperature.

1.2.3 Simulations of NMR spectra of [HP(μ-NTer)]$_2$

Technically, the NMR simulations of the expected line-shape after parahydrogen addition to [P(μ-NTer)]$_2$ were done by using numerical propagation of spin density matrix in Matlab. The density matrix obtained by averaging the H-H nuclear spin singlet state from parahydrogen in the scalar coupling environment of AA'XX' spin system of [HP(μ-NTer)]$_2$ was used as the initial hyperpolarized spin state after the adduct formation.[S4] The thermal spectra were calculated by setting the initial operator to be proportional to the I_z projection operator.[S5] The following J coupling parameters were used in the simulations in Hz: $J_{HH'}$ = 5.9, J_{HP} = 129.8, $J_{HP'}$ = 6.1 and $J_{PP'}$ = 21.7.[S2] It

should be noted that J_{HH} has a relatively large value for a long-range HH coupling constant through four chemical bonds. Likely, it is due to the specific structure of the biradicaloid adduct with H_2, which is relatively rigid. According to our preliminary computational studies, the main contribution to the J-coupling between the two hydrogens arises from the Fermi contact interaction. In addition, all molecular orbitals that are likely to partake in the coupling path show contributions to the P-H and P-N bonds. Therefore, it is hard to say whether the J-coupling is purely through-space or bond-mediated. It seems that a cooperative effect results in the large J_{HH} value. As this communication is focused on nuclear hyperpolarization effects, the structural features of $[HP(\mu\text{-}NTer)]_2$ will be addressed elsewhere.

1.2.4 Spin states of AA'XX' spin system of H-H and P-P pairs in $[HP(\mu\text{-}NTer)]_2$

The spin states of the resulting AA'XX' spin system is convenient to build in the representation of triplet and singlet nuclear spin pairs made out of ^1H (A nuclei) and ^{31}P (X nuclei) similarly like it is done in Ref.[S6] Since parahydrogen accommodates only the singlet nuclear spin state, it is obvious that all states with singlet H pairs in the AA'XX' (ST_+, ST_-, SS, ST_0) will be overpopulated after the parahydrogen addition. The last two states are not the eigenstates since they are premixed to T_0S and T_0T_0 states by the coupling network. Therefore, these latter states should be overpopulated as well, giving altogether six possible states (ST_+, ST_-, ST_0, SS, T_0S, T_0T_0). Consequently, there is no direct way for overpopulation of the triplet state of H pairs via the scalar coupling, meaning that states like T_+T_+ and T_-T_- should not contribute to the hyperpolarization observed in NMR spectra.

Figure S3 (bottom traces) shows theoretically that artificial overpopulations of T_+ or T_- states of H-H pairs give rise to the resonances that contribute to the most intense lines in the thermal ^1H spectrum (top trace) of AA'XX' spin system of $[HP(\mu\text{-}NTer)]_2$. There are altogether four of such states because P-P pairs can be also either in T_+ or in T_- to observe contributions to those lines. In contrast, according to the theory there is no contribution to these lines from the S state of the H-H pair. The lines are visible in the hyperpolarization experiment (Figure 1a), in spite of the inability of the scalar coupling network to generate singlet-triplet transitions. Thus, there should be some mechanism other than scalar coupling which leads to the overpopulation of T_+ and T_- states of the H-H pair. Likely, this observation tells about a complex relaxation dynamics leading to the singlet-triplet transitions.

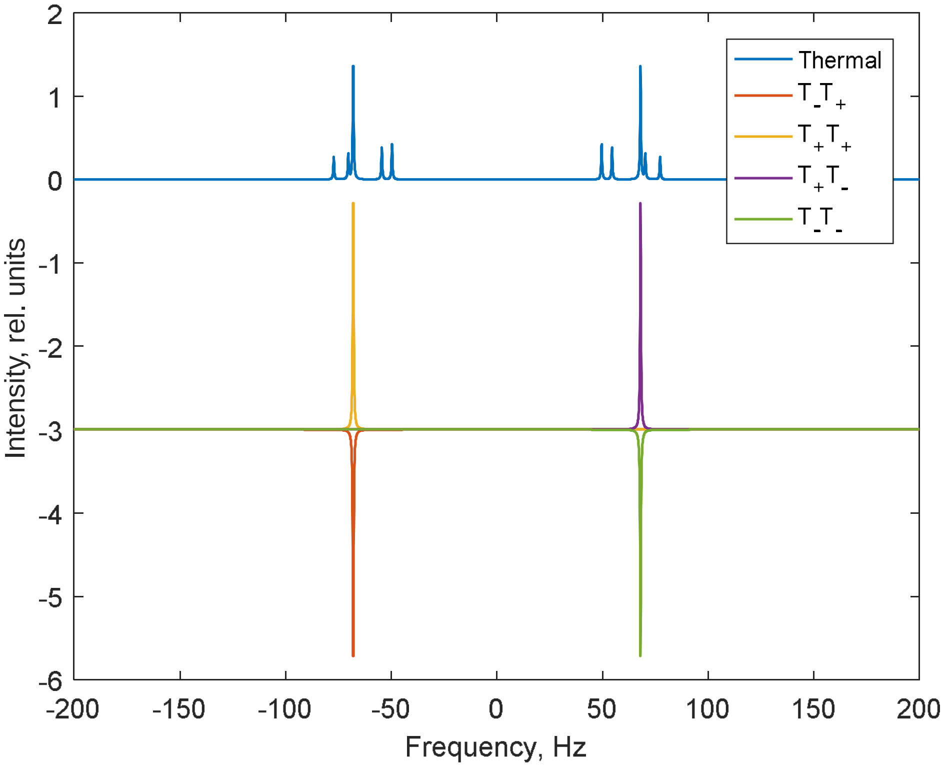

Figure S3. A simulated thermal ^{1}H NMR multiplet corresponding to H_2 originating atoms in [HP(μ-NTer)]$_2$ adduct (blue trace) and all possible non-zero contributions of the AA'XX' spin states to the multiplet's most intense transition lines (red, yellow, violet, green traces). See the legend for the contributing spin states.

1.2.5 Kinetic measurements

The measurements of kinetic constants were performed using spin saturation transfer method[S7] on a Bruker Avance III 400 MHz spectrometer. A heavy-wall 5 mm NMR tube equipped with a tight plug was used in the experiments. The sample was prepared by charging the tube with [P(μ-NTer)]$_2$ solution (~0.04 M) and 5 bar of normal (thermal) H_2. After the solution changed the color from dark orange to hardly visible yellow, the excess pressure was released by short opening of the cap. ^{1}H decoupling (WALTZ16) was used for all acquisitions. The thermally polarized ^{31}P signals (ca. 194 ppm) were used to determine the rate constants for the dissociation of [HP(μ-NTer)]$_2$ adducts into [P(μ-NTer)]$_2$ and H_2 (k_{dis}). The constants were calculated from the ratios of the signals in the normal spectra and in the spectra acquired with presaturation of the [P(μ-NTer)]$_2$ ^{31}P resonance at ca. 279 ppm. The resonance was presaturated in a continuous wave mode for 8 s. To determine k_{dis}, the following expression was used: $k_{dis} = (S_n/S_s - 1)/T_1$, in which S_n is the signal intensity in the normal spectrum, S_s is the signal intensity in the spectrum with presaturation, and T_1 (ca. 1 s) is the relaxation time of ^{31}P in [HP(μ-NTer)]$_2$.

1.2.6 SLIC spectra

The spin-lock induced crossing (SLIC)[S8] tests were done by applying a single RF pulse of weak power on the ^{31}P resonance frequency (121.5183837 MHz, ca. 194 ppm) after the parahydrogen bubbling. The employed NMR pulse sequence is shown in Scheme S1. The optimized values for the length and the power level were estimated by using numerical simulations. For example, Figure 4a shows the ^{1}H decoupled (WALTZ16) ^{31}P NMR spectrum acquired after applying 42 ms ^{31}P pulse of 63 Hz RF field amplitude. The small “satellite” signals in this spectrum is a manifestation of the non-ideality of the ^{1}H decoupling. The corresponding ^{1}H decoupled ^{31}P NMR spectrum obtained after the relaxation is shown in Figure 4b for comparison.

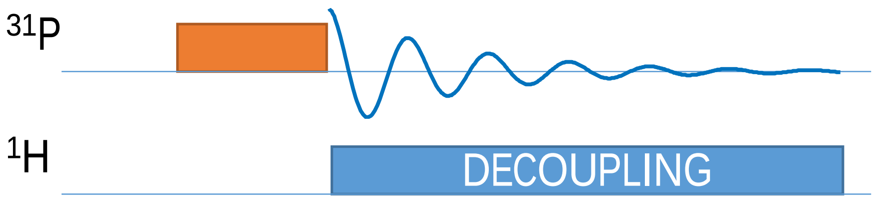

Scheme S1. Pulse sequence employed to perform ^{31}P SLIC experiments with ^{1}H decoupling.

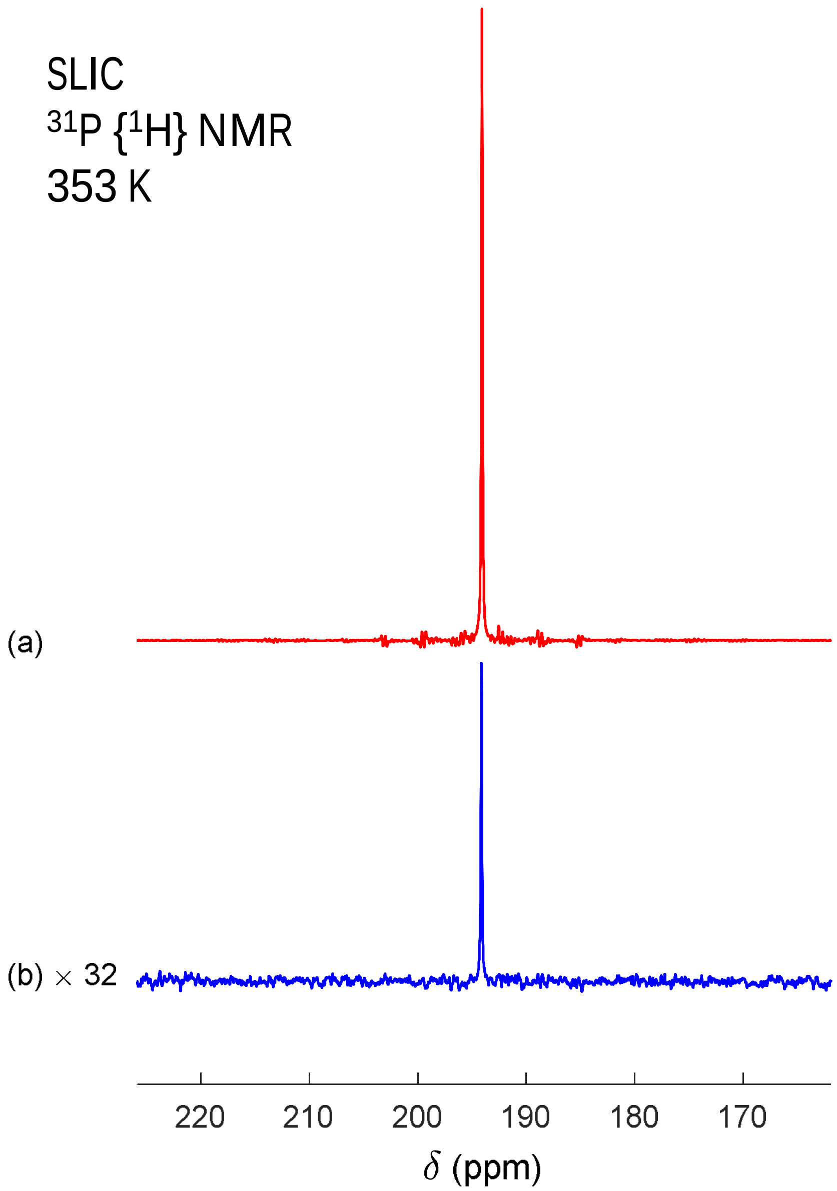

Figure S4. (a) Experimental ^{1}H decoupled ^{31}P NMR spectrum obtained by applying a weak-power (63 Hz RF field amplitude) 42 ms pulse after parahydrogen bubbling. (b) ^{1}H decoupled ^{31}P NMR spectrum obtained by using a hard π/4-pulse after the relaxation of spins to thermal equilibrium. The spectrum (b) is multiplied by a factor of 32 relative to spectrum (a). The small "satellite" signals in (a) are artifacts of non-ideal ^{1}H decoupling.

2 Computational details

Electronic structure computations were carried out using Gaussian09[S9] and ORCA 4.0.1.[S10,11] To estimate the activation barrier of H_2 release, the structures of the model compounds *cis*-[HP(μ-NPh)]$_2$, *trans*-[HP(μ-NPh)]$_2$, [H···P(μ-NPh)]$_2$ (transition state), [P(μ-NPh)]$_2$·H_2 (van-der-Waals complex), [P(μ-NPh)]$_2$, and H_2 were optimized at the PBE-D3(BJ)/def2-TZVP level of theory.[S12–16] All structures were confirmed as minima or transition states by frequency analyses. The optimized structures were used for single-point DLPNO-CCSD(T)/aug-cc-pVTZ calculations[S17–23] to obtain more reliable estimates of the electronic energies. The single point

energies were then added to the thermal corrections obtained from the frequency analyses to estimate the enthalpies *H* and Gibbs free energies *G* (Table S1).

Table S1. Calculated energies.

	PG	N_{im}	PBE-D3(BJ) E_{tot}	PBE-D3(BJ) ΔH_{corr}	PBE-D3(BJ) ΔG_{corr}	DLPNO-CCSD(T) E_{tot}	DLPNO-CCSD(T) $H°_{298K}$[a]	DLPNO-CCSD(T) $G°_{298K}$[b]
H_2	$D_{\infty h}$	0	−1.166	0.013	−0.002	−1.173	−1.159	−1.174
[P(μ-NPh)]$_2$	C_{2h}	0	−1254.750	0.204	0.146	−1253.616	−1253.412	−1253.470
[P(μ-NPh)]$_2$·H_2	C_s	0	−1255.921	0.219	0.157	−1254.793	−1254.574	−1254.636
[H···P(μ-NPh)]$_2$	C_s	1	−1255.909	0.217	0.158	−1254.773	−1254.556	−1254.615
cis-[HP(μ-NPh)]$_2$	C_{2v}	0	−1255.938	0.220	0.158	−1254.813	−1254.592	−1254.654
trans-[HP(μ-NPh)]$_2$	C_i	0	−1255.935	0.220	0.160	−1254.810	−1254.589	−1254.650

[a] Estimated by E_{tot}(DLPNO-CCSD(T)) + ΔH_{corr}(PBE-D3(BJ)).
[b] Estimated by E_{tot}(DLPNO-CCSD(T)) + ΔG_{corr}(PBE-D3(BJ)).

Hence, the activation barrier for H_2 release amounts to $\Delta H^‡$ = 22.6 and $\Delta G^‡$ = 24.4 kcal/mol, in good agreement with the experimental values. The activation barrier for the reverse reaction, i.e. addition of H_2 to the biradical, is much smaller ($\Delta H^‡$ = 9.7, $\Delta G^‡$ =18.1 kcal/mol), explaining the quick reaction at ambient temperature. The addition of H_2 is an exothermic and exergonic process ($\Delta H°$ = −12.9, $\Delta G°$ = −6.3 kcal/mol). Notably, the *trans* isomer of [HP(μ-NPh)]$_2$ is thermodynamically only slightly less favoured than the *cis* isomer ($\Delta G°$ = 2.7 kcal/mol). Thus, the exclusive formation of the *cis* isomer is a kinetic effect.

3 References

S1. T. Beweries, R. Kuzora, U. Rosenthal, A. Schulz and A. Villinger, *Angew. Chem. Int. Ed.*, 2011, **50**, 8974-8978.

S2. A. Hinz, A. Schulz and A. Villinger, *Angew. Chem. Int. Ed.*, 2016, **55**, 12214-12218.

S3. V. V. Zhivonitko, K. Sorochkina, K. Chernichenko, B. Kotai, T. Foldes, I. Papai, V.-V. Telkki, T. Repo and I. Koptyug, *Phys. Chem. Chem. Phys.*, 2016, **18**, 27784-27795.

S4. C. R. Bowers, in *Encyclopedia of Nuclear Magnetic Resonance*, eds. D. M. Grant and R. K. Harris, Wiley, Chichester, 2002, vol. 9, ch. Chapter, pp. 750-769.

S5. M. H. Levitt, *Spin dynamics: basics of nuclear magnetic resonance*, John Wiley & Sons, Chichester, England; Hoboken, NJ, 2nd edn., 2008.

S6. Y. Feng, T. Theis, T.-L. Wu, K. Claytor and W. S. Warren, *J. Chem. Phys.*, 2014, **141**, 134307.

S7. H. Friebolin, *Basic one- and two-dimensional NMR spectroscopy*, WILEY-VCH, Weinheim, 5th edn., 2011.

S8. S. J. DeVience, R. L. Walsworth and M. S. Rosen, *Phys. Rev. Lett.*, 2013, **111**, 173002.

S9. *Gaussian 09, Revision E.01*, M. J. Frisch, G. W. Trucks, H. B. Schlegel, G. E. Scuseria, M. A. Robb, J. R. Cheeseman, G. Scalmani, V. Barone, B. Mennucci, G. A. Peterson, H. Nakatsuji, M. Caricato, X. Li, H. P. Hratchian, A. F. Izmaylov, J. Bloino, G. Zheng, J. L. Sonnenberg, M. Hada, M. Ehara, K. Toyota, R. Fukuda, J. Hasegawa, M. Ishida, T. Nakajima, Y. Honda, O. Kitao, H. Nakai, T. Vreven, J. A. Montgomery Jr., J. E. Peralta, F. Ogliaro, M. Bearpark, J. J. Heyd, E. Brothers, K. N. Kudin, V. N. Staroverov, T. Keith, R. Kobayashi, J. Normand, K. Raghavachari, A. Rendell, J. C. Burant, S. S. Iyengar, J. Tomasi, M. Cossi, N. Rega, J. M. Millam, M. Klene, J. E. Know, J. B. Cross, V. Bakken, C. Adamo, J. Jaramillo, R. Gomperts, R. E. Stratmann, O. Yazyev, A. J. Austin, R. Cammi, C. Pomelli, J. W. Ochterski, R. L. Martin, K. Morokuma, V. G. Zakrezewski, G. A. Voth, P. Salvador, J. J. Dannenberg, S. Dapprich, A. D. Daniels, O. Farkas, J. B. Foresman, J. V Ortiz, J. Cioslowski and D. J. Fox, Gaussian, Inc., Wallingford CT, **2013**.

S10. F. Neese, *WIREs Comput. Mol. Sci.*, 2012, **2**, 73–78.

S11. F. Neese, *WIREs Comput. Mol. Sci.*, 2018, **8**, e1327.

S12. J. P. Perdew, K. Burke, M. Ernzerhof, *Phys. Rev. Lett.*, 1996, **77**, 3865–3868.

S13. J. P. Perdew, K. Burke, M. Ernzerhof, *Phys. Rev. Lett.*, 1997, **78**, 1396–1396.

S14. S. Grimme, J. Antony, S. Ehrlich, H. Krieg, *J. Chem. Phys.*, 2010, **132**, 154104.

S15. S. Grimme, S. Ehrlich, L. Goerigk, *J. Comput. Chem.*, 2011, **32**, 1456–1465.

S16. F. Weigend, R. Ahlrichs, *Phys. Chem. Chem. Phys.*, 2005, **7**, 3297–305.

S17. C. Riplinger, F. Neese, *J. Chem. Phys.*, 2013, **138**, 034106.

S18. D. G. Liakos, M. Sparta, M. K. Kesharwani, J. M. L. Martin, F. Neese, *J. Chem. Theory Comput.*, 2015, **11**, 1525–1539.

S19. C. Riplinger, P. Pinski, U. Becker, E. F. Valeev, F. Neese, *J. Chem. Phys.*, 2016, **144**, 024109.

S20. T. H. Dunning Jr., *J. Chem. Phys.*, 1989, **90**, 1007–1023.

S21. R. A. Kendall, T. H. Dunning Jr., R. J. Harrison, *J. Chem. Phys.*, 1992, **96**, 6796–6806.

S22. D. E. Woon, T. H. Dunning Jr., *J. Chem. Phys.*, 1993, **98**, 1358–1371.

S23. A. K. Wilson, T. van Mourik, T. H. Dunning Jr., *J. Mol. Struct.: THEOCHEM*, 1996, **388**, 339–349.
